# Structural Barriers to Primary Care Among Sex Workers: Findings from a Community- Based Cohort in Vancouver, Canada (2014–2021)

**DOI:** 10.21203/rs.3.rs-4802645/v1

**Published:** 2024-08-28

**Authors:** Miriam TH Harris, Kate Shannon, Andrea Krüsi, Haoxuan Zhou, Shira M Goldenberg

**Affiliations:** Boston Medical Center, One Boston Medical Center Place; University of British Columbia; University of British Columbia; University of British Columbia; San Diego State University

**Keywords:** women, sex work, primary care, violence, im/migration, barriers, facilitators

## Abstract

**Background:**

Due to structural marginalization, sex workers experience health inequities including a high prevalence of sexually transmitted and blood-borne infections, mental health disorders, trauma, and substance use, alongside a multitude of barriers to HIV and substance use services. Given limited evidence on sex workers’ broader primary healthcare access, we aimed to examine structural factors associated with primary care access among sex workers over a 7-year period.

**Methods:**

Data were derived from An Evaluation of Sex Workers Health Access (AESHA), a community-based open prospective cohort of women (cis and trans) sex workers in Metro Vancouver, from 2014 to 2021. Descriptive statistics were used to summarize the proportion of primary care use in the past six months and to assess primary care trends over time from 2014–2021. We used multivariate logistic regression with generalized estimating equations (GEE) to identify structural factors associated with primary care access (seeing a family doctor in the last six months), after adjusting for confounders.

**Results:**

Amongst 646 participants, most (87.4%) accessed primary care at some point during the study period, and primary care use in the in the last 6 months was relatively stable (ranging from 60–78%) across each follow-up period. At first available observation, participants faced a high burden of sexually transmitted and blood-borne infections (STBBIs) (48.0%, 11.5%, and 10.4% were HCV, HIV, or STI seropositive, respectively), 56.8% were diagnosed with a mental health disorder, 8.1% had recently overdosed, and 14.7% were recently hospitalized. In multivariable GEE analysis, exposure to intimate partner violence was associated with reduced access to primary care (Adjusted odds ratios (AOR) 0.63, 95% Confidence interval (CI): 0.49 – 0.82), and limited English fluency was marginally associated (AOR 0.76 CI: 0.51 – 1.14).

**Conclusions:**

This study characterized primary care access and its structural determinants among sex workers over 7-years. Participants faced a high burden of STBBIs and other health disparities, and a proportion faced gaps in primary care access. Scale-up of trauma-informed, culturally and linguistically tailored, sex worker-friendly primary care models are needed, alongside structural interventions to decriminalize and destigmatize sex work and substance use.

## Introduction

Due to structural marginalization and criminalization,x sex workers experience severe health inequities, including a high prevalence of sexually transmitted and blood-borne infections (STBBI), mental health disorders, trauma, and substance use (1, 2, 2–4). High-quality primary care that is accessible, timely, patient-focused, and comprehensive, is well positioned to address the unmet health care needs of sex workers (5, 6). Primary care providers are ideally positioned to deliver wrap-around health services to patients with multiple and often complex and competing health and social priorities (7, 8). Despite the promise of primary care for addressing sex workers’ unmet health needs, there is a paucity of studies assessing primary care engagement in this population, with most existing research focusing HIV, STIs, and substance use related services.

Primary care plays a particularly critical role in settings like Canada, where the majority of Canadians report seeing their family doctor almost exclusively for their medical care (9, 10). Primary care models that are community based, whose staff reflect the population they aim to serve (e.g., lived experiences, shared language), and are low barrier facilitate uptake among marginalized populations (11–14). Studies show that other marginalized populations, for example people living with HIV, are more likely to receive preventative health screening and have fewer hospitalizations when their medical care was predominately delivered by a family physician, compared to that of an HIV specialist (7). However, research on barriers and facilitators to health services among sex workers has largely focused on access to HIV and substances use services (3, 15, 16). Existing evidence suggests that structural factors such as criminalization, policing, violence, im/migration, stigma, housing instability are associated with barriers to HIV and substance use service prevention, treatment, and access to care (16–22), especially among sex workers who use criminalized substances or who have a mental health diagnosis (23, 24).

Given the high prevalence unmet health needs among sex workers and the potential for primary care to address these, it is important to examine determinants of primary care engagement among this population. Previous studies assessing HIV and substance use service use among sex workers demonstrate the significance of structural factors in heath service utilization however there is limited data on primary engagement. Therefore, this study aims to address this gap by assessing determents of primary care use amongst sex workers.

## Methods

### Aim

We aimed to examine the association between structural factors with primary care use amongst a community-based cohort of sex workers from Vancouver, Canada over 7-years.

### Study design

Data were derived from an open community-based cohort of women sex workers, An Evaluation of Sex Workers Health Access (AESHA), which initiated recruitment in 2010. As previously described (25), cis and trans women^1^ who engaged in sex work (exchanging sex for money) in the past 30 days, aged 14 and older, who were able to provide informed consent were eligible to participate. AESHA activities were established in collaboration with community-based sex work agencies and continues to work with a Community Advisory Board, with representatives from more than 15 community agencies (26). Mapping of outdoor/public sex work locations and indoor sex work venues was used to facilitate time-location sampling to recruit participants through outreach across Metro Vancouver area, complemented by online outreach to sex workers working in online solicitation spaces. The recruitment rate was ~85% (primary reason for nonparticipation was a lack of active sex work engagement). All participants provided written informed consent prior to study enrollment.

At enrolment and semi-annually, participants completed interviewer-administered questionnaires, conducted by a trained interviewer with extensive community and/or lived experience. After appropriate pretest counseling, Biolytical INSTI (Biolytical Laboratories Inc, Richmond, BC) rapid tests were offered for HIV screening. Reactive tests were confirmed by blood draw and Western blot testing at the British Columbia Centre for Disease Control. Urine samples were collected for gonorrhea and chlamydia, and blood samples for syphilis, hepatitis C virus (HCV) antibody, and HCV viremia testing. All participants received posttest counseling and those diagnosed with sexually transmitted infections (STIs) were provided treatment by an onsite study nurse and appropriate referrals were provided for new HIV and HCV diagnoses. The questionnaire captured demographic data, substance use patterns, social and interpersonal factors (e.g., condom use and negotiation, social cohesion), structural factors (e.g., experiences of violence, sex work environment, experiences of criminalization), and service utilization experiences (e.g., substance use, sexual health, and primary care). Participants received an honorarium of $65 CAD at each visit. The study holds ethical approvals from the Providence Health Care/University of British Columbia Research Ethics Board. The present analysis includes all AESHA participants who completed a baseline and at least one follow-up interview between 2014–2021 and who provided a valid response to the primary outcome variable (primary care use, last 6 months). The study was restricted to 2014 onwards as this is when the primary care and some structural factor questions were added to the AESHA questionnaire.

### Outcome variable

The primary outcome variable of primary care use was defined as responding “yes” to the question “have you ever seen a family doctor in the last six months”. Primary care use was a time-updated variable with occurrences within the past six months measured at enrolment and each semi-annual study visit. In Canada, primary care is delivered almost exclusively by family medicine doctors and less commonly family medicine nurse practitioners (27). “Family doctor” is the terminology used by most Canadians in lay discussions and research in reference to primary care in the Canadian setting (28).

### Structural explanatory variables

Several structural factors were selected as possible explanatory variables in our analyses. Structural variable selection was informed by existing literature on health service utilization among sex workers and other marginalized populations. Most structural variables were time-updated, measured semi-annually, save English fluency and immigration status which were time-fixed from baseline.

To assess gender-based and workplace violence, we included exposure to intimate partner violence (measured as moderate to severe physical or sexual intimate partner violence using the World Health Organization standardized intimate partner violence scale (29), yes vs no/or no intimate partner), and violence when doing sex work (defined as being abducted/kidnapped, sexually assaulted or attempted sexual assault, raped, strangled, physically assaulted/beaten, locked/trapped in a car, thrown out of moving car, assaulted with weapon, drugged, or trapped in room/ hotel/ housing etc, yes vs no/or not doing sex work). To capture im/migration experiences we explored several variables including having limited English fluency (defined as being not very comfortable, uncomfortable, or very uncomfortable with speaking English), having precarious immigration status (defined as reporting being a temporary resident, a permanent resident, having no documents, expired documents, or other, yes vs no), and lacking health care coverage (yes vs no). To capture the impact of stigma we included health care stigma experiences, defined as reporting being denied health services or, maltreatment in health settings, or overhearing derogatory gossip about sex work in health settings (yes vs no). To capture housing, we included being unstably housed (defined as living in an single room occupancy hotel, staying with parents/family/relatives, supportive housing, or other, yes vs no). To capture factors related substance use and sex work criminalization we included incarceration (yes vs no), and experiencing policing harassment when doing sex work (defined as being told by police to move, stopped, searched, followed, being moved elsewhere to work, verbally harassed, repeatedly monitored, detained, physically assaulted, drug equipment taken, condoms taken, searched for condoms, other property taken, propositioned to exchange sex, or coerced into providing sexual favors by the police, yes vs no).

### Confounder variables

Based on existing literature, potential confounders were selected that we hypothesized were related to primary care use and the above structural factors. These included time-fixed demographic variables of minority sexual orientation (defined as identifying as gay, lesbian, bisexual, asexual, queer, Indigenous two-spirit, and/or other non-heterosexual identities, yes vs no), gender minority (cis vs trans women, including transgender women, transexual women and other transfeminine identities) and racialization, defined as White, Indigenous (inclusive of First Nations, Inuit, Metis, or Inuit peoples), and Women of Colour (Asian, Black, Latinx) (30,31). Given the low proportion of participants who identified as Black in our sample (consistent with the Black population of British Columbia (<2%), we jointly examined Black and Women of Color to examine effects of racism among racialized women. Age, as continuous variable, was also included. HCV, HIV, STI serostatus were assessed based on lab test results. Other potential confounders included mental health diagnosis (time-varying, yes vs no), as well as time-varying measures of alcohol use (none vs less than daily vs daily), injection drug use (yes vs no), nonfatal overdose (yes vs no), and hospitalization (yes vs no) in the prior 6 months.

### Statistical analyses

First, we stratified participant characteristics by primary care use in the last six months at their first available observation and reported these as counts and percentages for binary variables and medians and interquartile range for continuous variables.

We used descriptive statistics to summarize the proportion of bi-annual interview visits where participates reported primary care use in the past six months during the study period. We assessed primary care use trends over time by calculating the proportion of the bi-annual interview visits involving primary care use during each calendar year from 2014 to 2021. To assess if there were any changes in primary care use over time we conducted a time-trend analysis. We used the Durbin-Watson test for autocorrelation to assess for any linear dependence between adjacent observations in our time series data.

Existing literature was used to guide initial selection of structural exposure variables. Structural variables that had a high degree of collinearity were excluded. Logistic regression was used to examine the association between structural variables and confounders with primary care use over the study period. Generalized estimating equations (GEE) with a logit-link function and exchangeable correlation matrix were used to account for repeated measurements amongst participants over time (32,33). Missing and intermittent data were handled using a complete case approach. Hypothesized confounders identified a priori based on their known association with healthcare access in the literature were considered in multivariable analysis. All statistical analyses were performed in SAS version 9.4 (SAS, Cary, NC). We reported two-sided p-values and 95% confidence intervals.

## Results

In total, 646 participants were included, who contributed 3881 observations over the seven-year period. The mean follow-up time was 6-study visits. Among the 646 included, there was missing primary care use data from three individuals. At first available observation of the 643 persons who reported primary care use, 562 (87.4%) reported using primary care at some point during the study. At participants’ first available observation, 387 (59.9%) used primary care at least once in the past six months. The median age was 39 years (IQR: 31.0–46.0), 31.9% identified as White, 43.0% as Indigenous, and 25.1% as Women of Colour (Table 1). Just under half (44.4%) reported a minority sexual orientation and 11.2% identified as a gender minority. Participants faced high prevalence of unmet healthcare needs: 48.0% were HCV seropositive, 11.5% were HIV seropositive, and 10.4% were STI positive based on lab data from the last 6 months. Mental health and substance use issues were also common. Over half (56.8%) of participants reported being diagnosed with a mental health disorder, and in the last six months 39.5% used alcohol, 41.5% reported injection drug use, 8.1% experienced a nonfatal overdose, and 14.7% had been hospitalized.

Participants faced a high degree of structural marginalization (Table 1). Data from first available observation showed violence was common where in the last six-months 12.7% reported exposure to intimate partner violence and 7.6% reported some form of violence or harassment while working. Related to im/migration experiences, 10.2% reported limited English Fluency, 24.6% were im/migrants to Canada, and 24.6% lacked health insurance. Over two-thirds were unstably housed. Experiences related to stigma and criminalization were also common, with 8.8% reporting healthcare stigma, 5.1% had been incarcerated, and 7.1% reported exposure to police harassment while doing sex work all within the last six-months.

**Figure 1:**
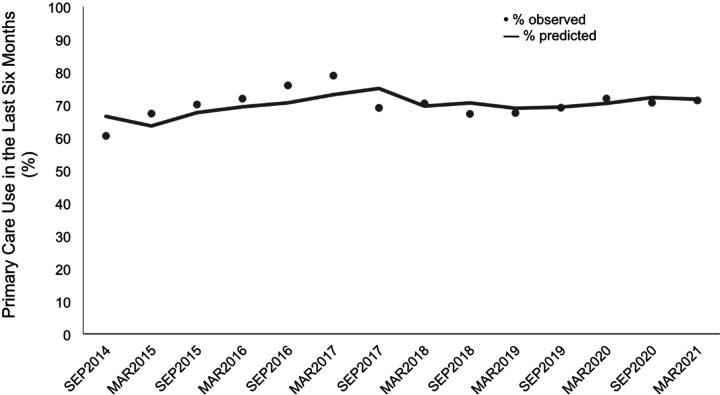
Period prevalence of primary care use at each six-month study period amongst a community-based cohort of women sex workers in Metro Vancouver, Canada, 2014–2021 (N = 646)

Figure 1 summarizes primary care use over time. Between 2014 to 2021 primary care use was documented to range from 60–79% at each follow-up period. Utilization was lowest (60.5%) in late 2014 and highest (78.6%) in the later part of 2016, though the time-trend analysis found no significant change in use over time. In total, 562/643 participants used primary care at some point during the study period.

In unadjusted analyses (Table 2), structural factors associated with reduced odds of structural factors associated with reduced odds of primary care use included exposure to intimate partner violence and limited English fluency. Other covariates that were associated with increased odds of primary care use included increasing age, minority sexual orientation, identifying as a gender minority, having a mental health disorder, and being hospitalized in the last six months. In the adjusted multivariable GEE analysis, exposure to intimate partner violence was independently associated with a reduced odds of primary care use (AOR: 0.63, 95% CI: 0.49 – 0.82, p=0.002) after adjustment for key confounders (age, minority sexual orientation, gender identity, racialization, mental health diagnosis, hospitalization, and overdose). Additionally, having limited English fluency was marginally associated with a reduced odds (AOR: 0.76 CI: 0.51 – 1.14, p=0.182) of primary care use.

## Discussion

This study provides some of the first epidemiologic data characterizing primary care use among women sex workers globally. In this 7-year prospective cohort study, sex workers faced high prevalence of health inequities related to STBBIs, mental health and nonfatal overdose, accompanied by a lack of ever using primary care among a proportion (~ 12.6%) of participants. After adjusting for confounders, women experiencing recent intimate partner violence faced 37% reduced odds of recent primary care use, and im/migrant women facing language barriers faced a 24% reduced odds of primary use, though this was only marginally significant (p = 0.182).

We found that most participants (87.4%) used primary care at least once throughout the study period. The study was conducted in a setting where provincially funded healthcare is provided to all residents without cost. However, health coverage is not extended to those with precarious im/migrants status and is thus not actually universal (34, 35). To mitigate barriers to primary care experienced by marginalized communities, Vancouver has invested in low-barrier primary care services, such as drop-in clinics, mobile outreach, and care embedded within shelter and housing programs. This may have facilitated access for participants in the Metro Vancouver area (36–38). However, participants in our study still had a high burden of unmet health care needs including a high prevalence of STBBIs, and a high rate of hospitalization, an important indicator of unmet primary healthcare needs and serious illness (39, 40). Findings from other studies suggest that such unmet health care needs may be related to barriers accessing needed health services within primary care due to service limitations, stigma, and language barriers (19, 35, 41–43). For example, women in our study had high rates of mental health diagnoses and substance use, but behavioral health and substance use services remain poorly integrated in primary care delivery (44–47). Criminalization of sex work and aspects of substance use, as well as internalized and institutional stigma, may also dimmish opportunities to address substance use and STBBIs within the context of primary care visits (19, 48–50).

Given the limitations of healthcare delivery for addressing broader structural drivers of the health inequities experienced by sex workers (16, 21, 51), structural interventions are crucially needed. Consistent with the literature, participants in our study experienced a high degree of structural marginalization, including intimate partner violence, housing instability, and criminalization. Violence against sex workers has been shown to be pervasive and rooted in both gender inequity and the criminalization of sex work and substance use (52–55). Importantly, we found that intimate-partner violence was associated with a reduced odds (AOR: 0.63, 95% CI: 0.49–0.82) of primary care use. This is consistent with research showing intimate-partner violence as a barrier to HIV and substance use services among sex workers and other structurally marginalized populations such as women who use substances (56–58).

Unfortunately, primary care is also often insufficiently equipped to identify and address gender-based violence which may exacerbate barriers. A 2022 qualitative meta-synthesis showed that primary care providers lacked knowledge, time, and resources to address violence (59). Violence services remain siloed from other health services and often structurally discriminate against sex workers (60). Thus, systemic structural changes and changes in primary care delivery are needed to reduce barriers, integrate violence services within primary care, and overcome gaps created by silos. For example, decriminalizing sex work would enhance environmental safety and promote access to health services by reducing the normalization and justification of violence against sex workers which criminalization promotes (21, 22, 61–63). Violence services must dismantle policies that discriminate against sex workers, such as refusing to accept women who use drugs or women who view sex work as a legitimate way of financially supporting themselves and their families (60). Additionally, investment in training and supports that facilitate sex worker-friendly trauma-informed approaches inclusive of addressing violence within primary care settings could further reduce barriers. Multi-component violence reduction interventions used in some HIV prevention and treatment services for sex workers offer models to integrating violence services within primary care (64–66).

Consistent with other studies, we found that limited English fluency was also associated with a reduced odds of primary care use. Though we found only marginal significance for this association these findings are of important public health significance. Prior literature identified English language fluency as a barrier to health services, particularly among im/migrants (41, 67). Language discordance between im/migrants and healthcare providers is identified as both a barrier to primary care access and diminished quality of care delivery, for example receiving lower rates of appropriate preventative health care services (5, 67). In addition to language barriers, im/migrants are also more likely to lack health insurance, access to culturally-responsive services, and experience disrespectful treatment by providers (34, 68). Such barriers and reduced health service quality can be exacerbated among sex workers due to the highly stigmatized and criminalized nature of sex work in Canada (69, 70). In addition to integrating culturally responsive translation services, which have been shown to diminish language barriers, on-going investments in low-barrier, sex-worker lead services are needed to address the complex intersecting factors of limited-English fluency, im/migration, and stigma mitigating health service engagement among sex workers (36, 71, 72).

Our findings must be interpreted within the study limitations. This study is based on observational data, and further research is needed to assess the pathways through which intimate partner violence and other structural factors influence primary care engagement for sex workers. There was missing longitudinal HIV, STI and HCV seropositivity data associated with interruptions in STBBI testing during COVID-19 research site closures. Further analyses examining intersectional impacts of marginalization related to gender minority status, sexual orientation, racialization, and im/migration status are also recommended. Our study relies on self-report data thus maybe subject to social desirability bias and underreporting of stigmatized issues and overreporting of positive health behaviors, such as our primary outcome of primary care use. However, the latter would attenuate our effect size towards the null. Additionally, our study looked at use alone, and did not explore quality of primary care experiences. Lastly, our study was focused on the experiences of sex workers who identified as women at baseline (cis or trans) in Vancouver, Canada, and thus did not sample for non-binary or male sex workers or those in other jurisdictions, limiting generalizability.

While primary care is well positioned to address women sex workers unmet healthcare needs our study highlights persistent structural barriers mitigating primary care engagement, thereby suggesting the critical importance of multi-level interventions targeting both policy and health service delivery environments. Our findings underscore the need for ongoing scale-up of trauma-informed, culturally, and linguistically tailored low-barrier primary care models. Community-based, sex-worker led services that include comprehensive sexual reproductive health care, substance use treatment, trauma and mental health care, and violence services are approaches that could enhance primary care use among sex workers. Scale-up of such sex-worker responsive services requires investment in alternate-care models alongside broader structural interventions to decriminalize and destigmatize sex work and substance use.

## Figures and Tables

**Table 1.** Baseline sample characteristics of sex workers in Metro Vancouver, Canada, stratified by primary care use, 2014–2021 (N = 646)

Characteristic	Total	Primary care use^a^	
	N (%)	N (%)	

		**Yes**	**No**
	646	387 (59.9%)	249 (38.5%)

**Demographic**			

Age (med, interquartile range)^a^	39 (31.0–46.0)	40 (32.0–46.0)	38 (30.0–46.0)

Minority sexual orientation^b^	287(44.4%)	176 (45.5%)	107 (43.0%)

Gender minority^b^	72 (11.1%)	47 (12.1%)	23 (9.2%)

Racialization^b^			
White	206 (31.9%)	120 (31.0%)	86 (34.5%)
Indigenous	278 (43.0%)	177 (45.7%)	94 (37.8%)
Black/Women of Color	162 (25.1%)	90 (23.3%)	69 (27.7%)

**Health**			

HCV seropositivity^c^	310 (48.0%)	210 (54.3%)	96 (38.6%)

HIV seropositivity^c^	74 (11.5%)	67 (17.3%)	7 (2.8%)

STI positivity^c^	67 (10.4%)	39 (10.8%)	26 (10.4%)

Mental health diagnosis^b^	367 (56.8%)	231 (59.7%)	131 (52.6%)

Alcohol use^a^			
None	384 (59.4%)	243 (62.8%)	133 (53.4%)
Less than daily	219 (33.9%)	126 (32.6%)	91 (36.6%)
Daily	36 (5.6%)	13 (3.4%)	23 (9.24%)

Injection drug use^a^	268 (41.5%)	160 (41.3%)	106 (42.6%)

Overdose^a^	52 (8.1%)	24 (6.2%)	26 (10.4%)

Hospitalized^a^	95 (14.7%)	63 (16.3%)	29 (11.7%)

**Structural**			

Intimate partner violence^a^	54 (8.4%)	24 (6.2%)	29 (11.7%)

Violence while working^a^	49 (7.6%)	24 (6.2%)	24 (9.6%)

Limited English Fluency^a^	66 (10.2%)	33 (8.5%)	32 (12.9%)

Im/migrant to Canada^a^	159 (24.6%)	85 (22.0%)	71 (28.5%)

No health insurance^a^	159 (24.6%)	90 (23.3%)	65 (26.1%)

Health care stigma^a^	57 (8.8%)	35 (9.0%)	21 (8.4%)

Unstably housed^a^	515 (79.7%)	312 (80.6)	197 (79.1)

Incarcerated^a^	33 (5.1%)	19 (4.9%)	13 (5.2%)

Police harassment while working^a^	46 (7.1%)	25 (6.4%)	20 (8.0%)

HCV; hepatitis C virus, HIV; human immunodeficiency virus, STI; sexually transmitted infection

Minority sexual orientation includes those who identified as lesbian, gay, bisexual, queer, and/or asexual

Gender minority included transgender women, transexual women and other transfeminine identities

Indigenous racial identities included First Nations, Inuit, & Metis. Women of Color included Black, Chinese/Taiwanese, Vietnamese, Korean, Japanese, Thai, Filipina, Indian, Pakistani, Bangladeshi, Sri Lankan, Latin American, Middle Eastern, or African

a In the last 6 months.

b In lifetime.

c Based on first available observation, there was 11% missing data for HCV serostatus, 20% for STI serostatus, and 9% for HIV serostatus

There was less than 5% missing data for all other characteristics.

**Table 2.** Unadjusted and adjusted generalized estimating equation (GEE) models of structural factors associated with primary care use in a cohort of women sex workers in Metro Vancouver, Canada, 2010–2021 (N = 646)

	Unadjusted odds ratio (95% CI)	Adjusted odds ratio (95% CI)

**Structural variables**		

Intimate partner violence^a^	**0.78 (0.65 – 0.95)**	**0.64 (0.49 – 0.82)**

Violence while working^a^	0.94 (0.70 – 1.25)	

Experienced health care stigma^a^	1.04 (0.81 – 1.35)	

Limited English fluency^a^	**0.59 (0.42 – 0.83)**	**0.76 (0.51 – 1.14)**

Unstably housed^a^	1.10 (0.90 – 1.33)	

Incarcerated^a^	1.09 (0.75 – 1.57)	

Police harassment while working^a^	0.85 (0.61 – 1.18)	

**Confounder variables**		

**Demographic**		

Age^a^	**1.03 (1.02 – 1.04)**	1.03 (1.02 – 1.04)

Minority sexual orientation^b^	**1.26 (1.00 – 1.59)**	1.11 (0.87 – 1.42)

Gender minority^b^	**1.60 (1.12 – 2.30)**	1.45 (0.98 – 2.15)

Racalization^b^		
White	-ref-	-ref-
Indigenous	1.04 (0.80 – 1.35)	1.12 (0.87 – 1.46)
Women of Color	0.70 (0.51 – 0.95)	0.83 (0.57 – 1.22)

**Health**		

Mental health disorder^b^	**1.33 (1.04 – 1.70)**	1.22 (0.93 – 1.60)

Hospitalized^a^	**1.34 (1.13 – 1.59)**	1.26 (1.04 – 1.54)

Alcohol use^a^		
None	-ref-	
Less than daily	1.06 (0.90 – 1.24)	
Daily	1.09 (0.83 – 1.42)	

Injection drug use^a^	0.90 (0.75 – 1.08)	

Overdose^a^	0.84 (0.68 – 1.05)	0.79 (0.62 – 1.01)

CI, confidence interval

Minority sexual orientation includes those who identified as lesbian, gay, bisexual, queer, and/or asexual

Indigenous racial identities included First Nations, Inuit, & Metis. Women of Color included Black, Chinese/Taiwanese, Vietnamese, Korean, Japanese, Thai, Filipina, Indian, Pakistani, Bangladeshi, Sri Lankan, Latin American, Middle Eastern, or African

a Time updated measure in the last six months.

b Time updated lifetime measure.

## Data Availability

The datasets used and/or analysed during the current study are available from the corresponding author on reasonable request.

## Footnotes

1. Eligibility was inclusive of cis women and other self-reported transfeminine identities at enrolment. As gender identity is fluid, some participants’ gender presentation differed throughout various times and aspects of their lives. For example, a participant may present as a woman/femme while interacting with sex work clients but identify as non-binary outside of work environments.

## Abbreviations

AESHA: An Evaluation of Sex Workers Health Access
GEE: generalized estimating equations
STBBI: sexually transmitted and blood-borne infection
AOR: adjusted odds ratio
CI: Confidence interval
HCV: hepatitis C virus
STI: sexually transmitted infection
